# In vitro effects of zinc on the cytokine production from peripheral blood mononuclear cells in patients with zinc allergy

**DOI:** 10.1186/s40064-015-1202-5

**Published:** 2015-08-08

**Authors:** Yoko Yoshihisa, Mati Ur Rehman, Takako Yamakoshi-Shibutani, Tadamichi Shimizu

**Affiliations:** Department of Dermatology, Graduate School of Medicine and Pharmaceutical Sciences, University of Toyama, Sugitani, Toyama, Japan

**Keywords:** Zinc, PBMCs, Cytokines

## Abstract

Metals, such as nickel, cobalt, chromium and zinc, are ubiquitous in the environment. Systemic reactions, including hand dermatitis and generalized eczematous reactions, can be caused by the dietary ingestion of metals. In this study, we aimed to determine whether the cytokine production from peripheral blood mononuclear cells (PBMCs) obtained from zinc allergy patients can be used as a sensitive marker to investigate zinc-allergic contact dermatitis. The diagnosis of sensitivity to metal was made based on the results of a metal patch test. The PBMCs were stimulated with various concentrations (5–100 μM) of zinc sulfate (ZnSO_4_) for 24 h. The culture supernatants were collected and analyzed using ELISA for measurement of the cytokine production. The levels of IFN-γ, TNF-α, IL-1β, IL-5, IL-13 and MIF were significantly higher in the zinc-allergic patients (n = 5) than in the healthy controls (n = 5) at 100 μM of ZnSO_4_ stimulation. Although, patch testing is considered as standard test to diagnose metal allergy but false-positive and -negative reactions may limit its use in conditions of existing dermatitis. Therefore, this study suggest that in support of patch testing the determination of cytokine production using PBMCs cultures would be helpful for making an early diagnosis of such conditions.

## Background

Modern day living and industrialization resulted in increased cutaneous exposure to metals like nickel, cobalt, chromium and zinc, which are ubiquitous in the environment. Cutaneous exposure to these metals caused increased incidence of metal allergies (Thyssen and Linneberg 2007). Metal allergies may lead to the pathogenesis of allergic contact dermatitis or systemic contact dermatitis. Electrophilic metals may ionize and react with proteins to form complexes, dendritic cells can easily identify such complexes, which results in sensitization (Jacob and Zapolanski 2008). It has been reported that cultures collected from peripheral blood mononuclear cells (PBMCs) of metal-allergic patients, showed a particular pattern of cytokine production, including both T helper type 1 (Th1) and T helper type 2 (Th2) cytokines, when stimulated with metals like nickel, cobalt and chromium (Falsafi et al. 2000; Minang and Arestom 2006). In addition, nickel-induced cytokine production from mononuclear cells in nickel-sensitive individuals and controls has also been reported (Borg et al. 2000).

Zinc is an important regulatory factor in the immune system and plays an important role in multiple aspects of the immune function, including maintaining the skin barrier to promoting lymphocyte maturation, activation and regulation (Cunningham-Rundles et al. 1990; Rink and Kirchne 2000). Previously, our group reported the induction of systemic contact dermatitis (SCD) by zinc allergies (Yanagi et al. 2006), in which we described the relationship between SCD and the production of cytokines induced by zinc. Contact dermatitis resulting from direct contact with an allergen is the most common and easiest form of metal allergy to identify.

In this study, we investigated the in vitro effects of zinc on the cytokine production from PBMCs obtained from zinc-allergic patients.

## Methods

### Materials

The following materials were obtained from commercial sources: Ficoll–Paque Plus from GE Healthcare Bio-Sciences AB (Uppsala, Sweden); RPMI 1640 and streptomycin from Sigma (St Louis, MO, USA); Zinc sulfate (ZnSO_4_) from Wako pure chemical industries (Osaka, Japan); fetal bovine serum from Gibco Co. (Grand Island, NY, USA); IFN-γ, TNF-α, IL-1β, IL-5, IL-13 and MIF ELISA kits from R&D Systems (Minneapolis). All other chemicals were of reagent grade.

### Subjects and study design

In order to investigate the influence of zinc-specific cytokine secretion by PBMCs on the clinical outcome (patch test score) in zinc-allergic patients with a history of contact dermatitis and lichen planus were included in this study. The diagnosis of zinc allergy was confirmed based on a positive patch test reaction to zinc chloride. Test sites were evaluated according to the criteria of the International Contact Dermatitis Research Group (ICDRG). Five zinc-allergic patients (three males and two females), with an age range of 41–65 years (mean age 54.0 years) who attended our clinic between October 2011 and May 2015, and five healthy volunteers as control (four males and one female), with an age range of 33–50 years (mean age 39.6 years), were enrolled in this study (Table 1). The recruited subjects in this study were only five, with different age and gender because the cases of zinc allergy are not very frequent. Zinc-allergic patients were not sensitized to other metals including nickel or cobalt. All patients have provided their informed consent for participation, in compliance with all Principles of the Declaration of Helsinki. This study was approved by the Medical Ethics Committee of the University of Toyama, Toyama, Japan.

**Table 1:** Subjects profile and patch test reactivity to zinc

Subject no.	Sex	Age (year)	Patch test	Clinical history (duration)	Drug treatment
Patients					
1	F	59	+	Lichen planus (9 months)	No
2	M	41	++	Contact dermatitis (4 years)	Steroid ointment
3	F	52	+	Contact dermatitis (2 weeks)	No
4	M	77	++	Contact dermatitis (1 month)	No
5	F	65	++	Lichen planus (1 year)	No
Controls					
1	M	50	–	–	–
2	M	42	–	–	–
3	M	37	–	–	–
4	F	33	–	–	–
5	F	36	–	–	–

### Human PBMC isolation

PBMCs obtained from the healthy controls (n = 5) and patients with zinc allergy (n = 5) were prepared in heparinized blood using Ficoll–Paque plus (GE Healthcare Bio-Sciences AB, Uppsala, Sweden) density gradient centrifugation. The PBMC layer was washed three times with sterile PBS. The PBMCs (2 × 10^6^ cells/mL) were cultured in RPMI 1640 (Sigma-Aldrich Co.) containing streptomycin (50 μg/mL, Sigma-Aldrich Co.) and 5 % heat-inactivated fetal bovine serum (Gibco Co., Grand Island, NY, USA) in six-well plates at 37 °C in a humidified atmosphere of 5 % carbon dioxide. The cells were either stimulated with various concentration of ZnSO_4_ (ranging from 5 to 100 μM) or left unstimulated. After 24 h of culture, the supernatants were collected and frozen until the ELISA assay. We sampled the patient’s PBMCs prior to administering treatment.

### Cytokine production

The supernatants of the cultured PBMCs were subjected to ELISA for measurement of the IFN-γ, TNF-α, IL-1β, IL-5, IL-13 and MIF levels. The absorbance was measured with a microplate reader.

### Statistical analysis

Differences between the various treatments were statistically tested using the Mann–Whitney U test. P values of <0.05 were considered to be statistically significant. The data in the figures are presented as the mean ± SE.

## Results

The allergic reaction to ZnSO_4_ was assessed using a patch test; the subject profiles and levels of reactivity to zinc are shown in Table 1. Strong reactivity to zinc was observed in the zinc-allergic patients (n = 5), whereas the healthy control subjects had no reaction to zinc (n = 5).

We examined the production of pro-inflammatory cytokines in each individual following the in vitro stimulation of PMBCs and measured the secretion of Th1 cytokines (IFN-γ, TNF-α and IL-1β), Th2 cytokines (IL-5 and IL-13) and MIF, which are secreted from both types of T helper cells. The levels of Th1 and Th2 cytokines were each significantly higher following zinc stimulation at a concentration of 50 and 100 μM in the zinc-allergic patients. On the other hand, these effects were not observed in the normal healthy control subjects upon zinc stimulation (Table 2). Taken together, these findings clearly show that zinc stimulation specifically increases both the Th1 and Th2 cytokine production from PMBCs in zinc-allergic patients, but not healthy controls.

**Table 2:** IFN-γ, TNF-α, IL-1β, IL-5, IL-13 and MIF from subjects’s PBMCs

Zn (μM)	IFN-γ (pg/mL)	TNF-α (pg/mL)	IL-1β (pg/mL)	IL-5 (pg/mL)	IL-13 (pg/mL)	MIF (ng/mL)
Patient 1						
0	1.7	1.4	1.3	0.3	<0.2	1.5
5	5.2	38.2	1.6	0.5	1.3	1.5
50	11.6	42.8	18.9	2.6	7.9	2.8
10	19.3	71.3	22.5	5.8	18.7	7.8
Patient 2						
0	1.3	<0.01	0.8	<0.2	<0.2	2.1
5	10.5	30.2	1.0	<0.2	3.6	3.2
50	17.2	50.5	20.3	3.7	10.6	5.4
100	25.3	88.8	24.2	6.9	16.3	8.0
Patient 3						
0	0.9	0.3	1.2	<0.2	0.3	5.3
50	9.8	15.2	1.7	0.3	5.5	6.8
50	12.3	13.8	20.1	3.8	14.6	6.2
100	20.5	19.1	26.6	9.2	2.3	6.9
Patient 4						
0	1.6	<0.01	0.9	<0.2	0.4	0.8
5	8.3	12.1	0.8	0.8	9.6	1.2
50	15.5	13.2	12.3	2.1	14.5	3.8
100	20.3	15.7	18.9	6.3	20.3	9.7
Patient 5						
0	0.7	0.4	<0.04	<0.2	<0.2	1.3
5	1.0	5.5	0.8	1.0	<0.2	2.4
50	5.6	11.2	18.6	11.5	13.2	3.2
100	13.3	35.0	23.5	20.7	19.7	7.0
Control 1						
0	1.2	<0.01	0.5	<0.2	0.9	1.1
5	1.6	<0.01	0.9	0.3	1.0	1.3
50	6.3	<0.01	1.5	0.5	1.3	3.8
100	8.5	<0.01	2.3	0.5	1.5	2.6
Control 2						
0	1.7	<0.01	1.1	<0.2	0.5	0.4
5	3.6	<0.01	1.5	0.4	0.8	0.3
50	4.9	<0.01	1.8	0.4	0.8	0.8
100	6.3	<0.01	1.8	0.8	1.2	0.6
Control 3						
0	1.8	<0.01	0.8	<0.2	0.6	0.2
5	3.2	<0.01	1.1	<0.2	0.6	1.1
50	5.1	<0.01	1.3	<0.2	1.0	0.4
100	7.2	<0.01	1.6	0.4	1.1	1.1
Control 4						
0	0.9	<0.01	1.0	0.1	0.8	1.4
5	2.6	<0.01	0.9	0.1	0.9	1.3
50	4.9	<0.01	1.1	0.1	0.9	1.0
100	7.8	<0.01	1.8	0.4	1.2	0.9
Control 5						
0	0.3	<0.01	<0.04	<0.2	<0.2	0.3
5	0.3	<0.01	<0.04	0.3	<0.2	0.5
50	0.7	<0.01	<0.04	1.0	<0.2	0.6
100	1.6	<0.01	0.4	0.3	<0.2	0.3

**Fig. 1:**
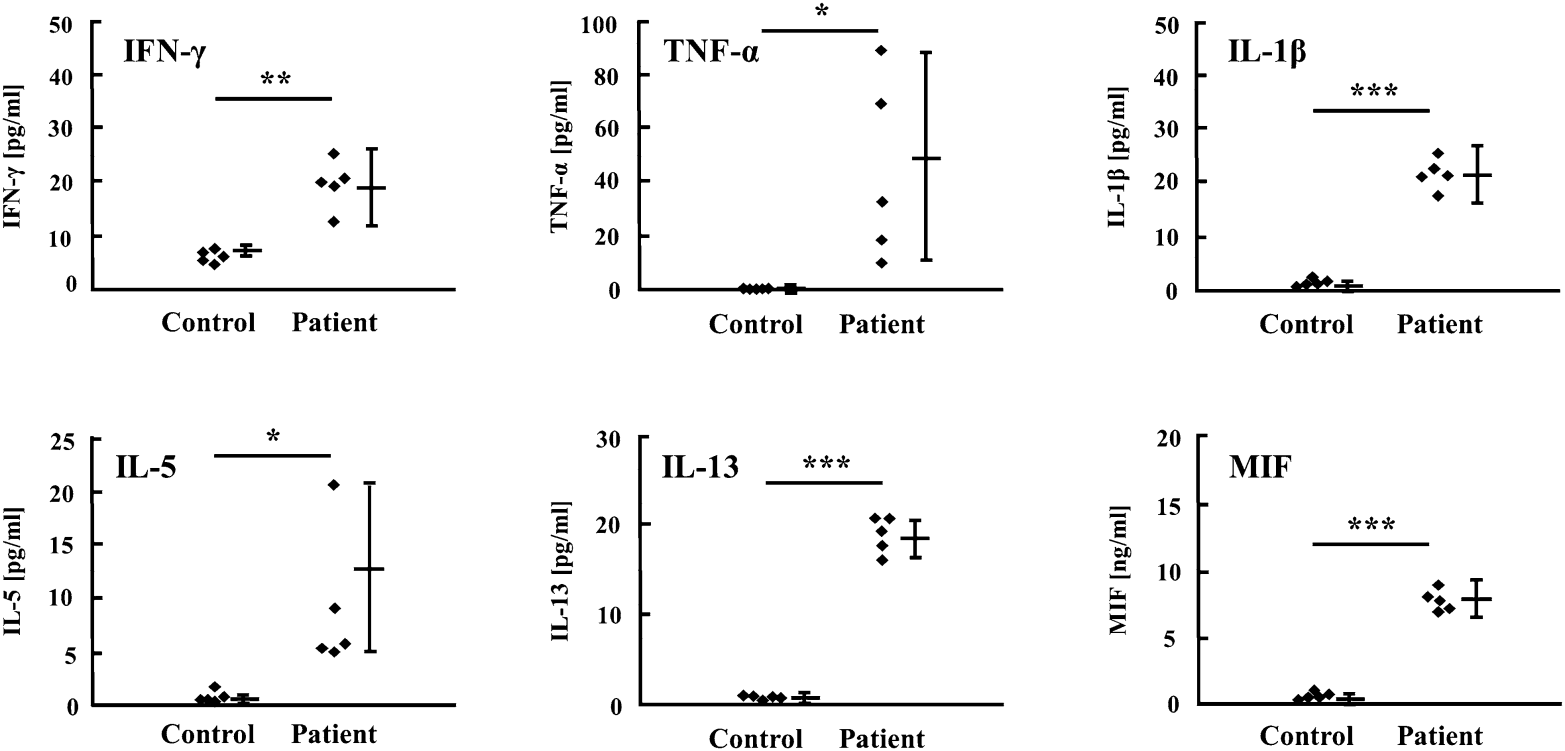
Cytokine induction by ZnSO_4_ in PBMCs. PBMCs obtained from patch test-positive or control subjects were stimulated for 24 h with 100 µM of ZnSO_4_. The IFN-γ, TNF-α, IL-1β, IL-5, IL-13 and MIF content in the cultured supernatants was analyzed using ELISA. The data in the figures are presented as the mean ± SE. *p < 0.05, **p < 0.005, ***p < 0.001.

Furthermore, the results of the statistical analysis of the cytokine values observed following stimulation with 100 μM of zinc support this observation. As shown in Fig. 1, stimulation with 100 μM of zinc induced a significant increase in the production of Th1 cytokines IFN-γ, IL-1β and TNF-α in the zinc-allergic patients compared to that observed in the healthy control subjects (**p < 0.005 for IFN-γ, *p < 0.05 for TNF-α and ***p < 0.001 for IL-1β). Similar effects were observed for Th2 cytokines and MIF (*p < 0.05 for IL-5, ***p < 0.001 for IL-13 and MIF). These results demonstrate that zinc stimulation resulted in significant differences between the two groups.

## Discussion

In our experimental system, we determined the cytokine profiles in zinc-allergic patients compared to those of healthy controls and monitored the cytokines levels in the PMBCs of both groups following exposure to ZnSO_4_ at different concentrations. We found that zinc stimulation resulted in significantly higher levels of cytokines in the patients than in the control subjects and that an elevated zinc level specifically increased both the Th1 and Th2 cytokine production in the PMBCs of the zinc-allergic patients, but not the healthy controls. These findings are consistent with those of our previous report and the observations of different groups that demonstrated increased levels of MIF, TNF-α and IL-1β from PMBCs in zinc-allergic patients compared to healthy controls (Yanagi et al. 2006). It has also been reported that zinc enhances the induction of TNF-α and IL-1β following stimulation of PMBCs with lipopolysaccharide (LPS) (Yanagi et al. 2006; Wellinghausen et al. 1996a). Furthermore, zinc stimulates monokine production from PMBCs in the serum and a LPS free cell culture system. It was also demonstrated that zinc increased monokine secretion more efficiently than other related divalent cations including cobalt, nickel and mercury. Therefore, it was suggested that the induction of cytokines in PMBCs was a possible mechanism of zinc-specific immune modulation (Wellinghausen et al. 1996b). The data obtained in the present study support the notion that determining the cytokine profiles using the in vitro stimulation of PBMCs with metal salts alone is a useful diagnostic method.

Zinc is an essential trace element and plays an important role in regulating different physiological functions as well as in immune system (Chang et al. 2006). It is become evident that zinc deficiency may cause increased susceptibility for different pathogens and very often the impaired immunologic functions in zinc-deficient subjects are controlled by providing zinc supplementation (Prasad 1995). Studies have also postulated the role of zinc other than immunological functions, because zinc has been regarded as a key factor for the activity of more than 300 enzymes (Prasad 1995; Vallee and Auld 1993). It has been observed that presence of zinc in the food items and in dental restoration, may enhance the severity of SCD symptoms (Yanagi et al. 2006; Yoshihisa and Shimizu 2012). Although the systemic allergic reactions with zinc are rare but dental eruptions due to zinc including oral lichen planus (Ido et al. 2002; Ito et al. 2012), palmoplantar pustulosis (Yanagi et al. 1050), and systemic dermatitis (Saito et al. 2010) have been reported previously. In most cases, the main causative factor was dental fillings containing zinc, and the patients were treated efficiently after removing the dental fillings or prescribing a zinc-restricted diet (Yanagi et al. 2006; Saito et al. 2010; Sakai et al. 2013). It has also been known that the toxicity and allergic capacity of the zinc depend on its speciation. Zinc sulphate is considered more toxic than zinc glutamate but the physiological and pharmacologic/toxicologic effects of zinc are concentration-dependent. It has been reported that the normal physiological serum concentration of zinc had no effect on the immunologic or biological functions of PBMCs in healthy subjects. On the other hand, a zinc concentration of higher than 100 μM was shown to cause decreased cell viability and stimulated cytokine expression at the concentration of at least 100 μM (Chang et al. 2006). In consistent with previous report, we investigated the effects 100 μM zinc sulphate, on the cytokine production in the PMBCs of suspected zinc-induced contact dermatitis patients.

Different diagnostic methods have been used to diagnose metal sensitivity, each of which have merits and demerits. Epicutaneous path testing is a primary tool for diagnosing allergens that cause allergic contact dermatitis. It has several advantages which includes less adverse reactions and can be easily employed without proper hospital surveillance. Therefore, patch testing has been regarded as a gold standard test for the determination of metal hypersensitivity but the actual accuracy of this method is solely achieved by the experience of observer (Yoshihisa and Shimizu 2012). In addition, false-positive and negative reactions may limit its use in conditions of existing dermatitis (Fisher and Rystedt 1985). In vitro tests, such as the lymphocyte stimulating test (LST), offer several advantages compared to patch testing in that they do not induce flare-up reactions or aggravate symptoms and can be efficiently used in clinical conditions in which patch testing is not recommended (Jensen et al. 2004). However, the use of LST as the sole diagnostic test for ruling out suspicion of a contact allergy has not been validated. It is very important to identify a diagnostic method for determining metal sensitivity at the early stage.

## Conclusion

In conclusion, along with patch testing, the determining of cytokine production using PBMCs cultures is a potentially promising in vitro method for identifying metal allergies and it may be helpful for making an early diagnosis of this condition.

## Abbreviations

PBMCs: peripheral blood mononuclear cells
SCD: systemic contact dermatitis
LST: lymphocyte stimulating test
LPS: lipopolysaccharide
